# Harold F. Mole

**Published:** 1918

**Authors:** 


### HAROLD F. MOLE.

We regret to have to record the death at his residence, 24 College Road, Clifton, of Mr. Harold F. Mole, F.R.C.S., in his 52nd year, from heart failure consequent on asthma, from which disability he had suffered for so many years.

He was a son of F. M. Mole, Esq., of Edgbaston, Birmingham, and owing largely to his tendency to asthma was sent as a boy to school in Clifton. He subsequently studied medicine at the Bristol Medical School and Royal Infirmary, and took the diplomas of L.R.C.P., M.R.C.S. in 1890, and two years later, after a course of study at St. Bartholomew's, London, he was admitted F.R.C.S.

While a student at the Royal Infirmary he was awarded the Tibbits Memorial Prize for practical surgery, and such was his popularity that in 1891, after holding the junior resident appointments, he was made Curator of the Museum, the post having been created by the medical staff in order to retain him as a colleague. From 1895 to 1902 he was in residence, first as House Physician and then as House Surgeon and Senior

Resident Officer, where his methodical habits, combined with his clinical acumen, rendered him not only an able administrator and a most reliable resident, but also a sound surgeon and a popular tutor.

In 1902 he was elected Assistant Surgeon to the Royal Infirmary, with charge of the aural clinic. His knowledge of otology soon became very thorough, owing to the keen interest he took in the work and to the experience he gained at the Royal Ear Hospital in London, where he studied the subject for six months prior to taking up the duties of his new post. In this connection he made a set of beautiful metallic casts of the labyrinth, which will in due course be mounted in the Museum of the Bristol University.

Considering how physically weak he was, Mr. Mole displayed a wonderful amount of energy. He acted as Secretary to the Bristol Medico-Chirurgical Society from 1903 to 1907, to the *Journal* of which Society he made several contributions of great interest and much value.

In 1909 he was made full Surgeon to the Bristol Royal Infirmary and clinical teacher in Surgery to the University until 1916, when ill-health compelled him to resign these offices. The physical strain of his work in the operating theatre often taxed his powers of endurance very severely, but it was characteristic of him that he never allowed this cause to interfere with the quality of his work, He countenanced no half-measures or compromises, whatever he undertook to do he did with the very best of his ability.

A large circle of friends has been deprived of a charming personality by his death, and the medical profession has lost a highly-talented member.

In 1913 Mr. Mole married Miss H. Holmes. He leaves a widow and three children, two sons and one daughter.

